# Novel Strategies for the Development of Healthier Meat and Meat Products and Determination of Their Quality Characteristics

**DOI:** 10.3390/foods10112578

**Published:** 2021-10-26

**Authors:** Claudia Ruiz-Capillas, Ana M. Herrero

**Affiliations:** Institute of Food Science, Technology and Nutrition (ICTAN-CSIC), José Antonio Novais 10, 28040 Madrid, Spain

Meat and meat products are very popular foods and widely accepted by consumers. However, the global production and consumption dynamics of meat and meat products have evolved rapidly as the result of changing lifestyles and nutritional ideologies among part of the population. As a result, it is important to address several different aspects regarding the quality of meat and meat products, particularly those having to do with nutrition (as relates to health), safety and sustainability. Meat and meat products contain essential components of the diet, providing a large number of nutrients such as protein of high biological value, fat, mineral vitamins with high bioavailability, etc. However, its consumption has been linked to some negative health consequences due to some of its components such as lipids, salt, additives, and others. Consumers now view meat products as less healthy and less attractive, and this makes them more selective in the products they consume, as they are increasingly aware of improving their health through the foods they consume. Health is one of the main criteria for consumers when choosing the type of food they consume. In addition, this diet-health-disease relationship, together with a higher life expectancy, has sparked public administrations to promote the development of healthier foods with the aim of improving quality of life.

At present, there is growing market demand for healthier and more functional foods, and this is one of the main drivers of new trends and innovations in the development of new healthy products, including those of meat origin. Strategies for the development of healthy meat products are based mainly on the reduction or elimination of unhealthy components, which may or may not be replaced by a healthy alternative. This is done while maintaining the same quality criteria demanded of traditional products in terms of their sensory, technological, nutritional, functional, safety and other characteristics.

Strategies for the development of healthier meat products can be applied at different stages in the farm to fork food chain in order to obtain healthy and high-quality products. The first stage in the development of healthy meat products occurs at the farm during breeding and animal production through both genetic and nutritional strategies. Through these strategies, the composition of meat and its derivatives are modified and optimized with a healthier profile. However, it must be considered that any manipulation at this level, for example of animal diet, has effects on production and the final quality of the meat.

Another stage in the farm to fork food chain in which meat products with a more beneficial composition can be developed is the reformulation and elaboration of meat products. This is one of the most common technological strategies used in the design and development of new healthier meat products as it has an immediate impact on food composition as opposed to genetic modification or changes in animal feed. This strategy can be quickly applied by the meat industry. Regarding the reformulation of meat and meat products, different strategies have been explored to optimize the composition of these products, make them healthier and bring them in line with health recommendations and nutritional guidelines promoted by public bodies in response to new consumer demands [1]. There are basically two meat product reformulation strategies: reduce or eliminate certain unhealthy ingredients (salt, nitrites, saturated fatty acids, etc.) and add healthy ingredients (natural antioxidants, polyunsaturated fatty acids, probiotics, prebiotics, vitamins, etc.), or combinations of the two, the latter strategy being the most common. The beneficial compounds added to meat products can be of different origins (plant, marine, etc.). A current trend that is attracting considerable attention is the use of plant-based ingredients (extracts, by products, waste material, etc.), as multifunctional compounds in meat products. This is due to the added nutritional value and bioactive compounds (minerals, vitamins, polyunsaturated acids, antioxidants, etc.) and also to technological interest (water and fat binding capacity, emulsifying and gelling properties, etc.). Among the different reformulation strategies, considerable attention has been paid to those that optimize the lipid content and profile of meat products to meet nutritional needs and adhere to health recommendations. The excessive consumption of fatty foods is associated with health problems such as obesity, diabetes and cardiovascular disease that are all very common in today’s society. The new lipid optimization strategies for the production of meat products seek to find or prepare ingredients similar to fat, such as analogues, imitators, mimetics, etc. One of today’s most popular trends is lipid structuring (organogels, bulking agents, structured emulsions, etc.) [1]. Strategies aimed at reducing ingredients such as salt or additives such as nitrates and nitrites have also been thoroughly studied.

Meat product processing, conservation and consumption conditions are also considered critical stages in the process of gaining or losing healthy properties since, to a greater or lesser extent, these factors will alter products’ components either by modifying or forming new compounds that may have positive or negative effects. This is the case, for example, of domestic practices once the product has been purchased. It is important to realize that once a healthier meat product has been developed, these beneficial properties must be maintained (farm to fork) so that it reaches the final consumer in optimal conditions and can have the beneficial effects intended.

The title of this Special Issue (SI) “Novel Strategies for the Development of Healthier Meat and Meat Products and Determination of Their Quality Characteristics” is based on all these considerations, our aim being to collect all new relevant information, which is of interest to all stakeholders (public institutions, livestock farmers, meat industry, technologists, researchers, teachers, students, etc.), and also to encourage researchers to search for new strategies to obtain healthier meat and meat products.

This special issue brings together 11 scientific contributions (2 reviews and 9 original research articles). It provides an interesting overview of the different strategies that can be applied at all stages from farm to fork; strategies that entail modifying meat and meat products to make them healthier and more sustainable. The SI also includes different meat and meat product quality control methods that have been developed.

Regarding strategies applied at the farm stage, a contribution on dietary manipulation is included, which studies the effects of algae meal supplements in feedlot lambs with competent reticular groove reflex (RGR) on growth performance, carcass traits and meat characteristics [2]. One of the main objectives of this strategy is to improve the quality of lamb meat by increasing omega-3 polyunsaturated fatty acid (PFA) levels. This is very interesting for the production of different meat products and for direct consumption as both are sources of omega-3 PFAs. In addition to this nutritional enhancement, the results of this study show that the addition of 2.5% seaweed to the diet of feedlot lambs with competent RGR has no detrimental effect on animal performance, carcass traits or quality characteristics of the meat (tenderness, colour, etc.).

Feng et al. [3] studied the quality characteristics (myofibril fragmentation index, total protein solubility, sarcoplasmic protein solubility, myofibrillar protein solubility, microstructure etc.) of different beef muscles (*semitendinosus*, *longissimus horacis*, *rhomboideus*, *gastrocnemius*, *infraspinatus*, *psoas major* and *biceps femoris*) during post-mortem ageing periods. The aim was to contribute new information to help improve and optimize the processing and storage of different cuts of beef in order to improve meat quality, mainly in terms of tenderness and acceptability. Both of these characteristics are critical indicators impacting people’s willingness to purchase meat, and are also an issue in the elaboration of meat products containing these muscles.

Several of the articles in this SI focus on reformulation strategies. For example, Mahachi et al. [4] studied the use of different types of fat (fat-tailed sheep tail and pork back fat) in the development of novel warthog cabanossi. The study mainly focused on the physicochemical, fatty acid and sensory attributes of warthog cabanossi. This study concluded that the combination of fat-tailed sheep ‘tail together with pork back fat could be an alternative to using only pork back fat to produce another variety of cabanossi of acceptable quality and the characteristic aroma and flavour but with some beneficial nutritional properties in terms of the n-3: n-6 ratio. This new product also gives consumers more options.

On the topic of reformulation, other authors have presented the use of mushrooms in the preparation of frankfurter [5] and nuggets [6] to reduce salt and fat in processed products and improve their nutritional profile. Both works focus on the use of plant by-products as functional food ingredients in meat products as these are a rich source of dietary fibre and various other bioactive compounds such as vitamins, minerals and polyphenols with strong antioxidant potential. Ceron-Guevara et al. [5] evaluated the effect that partially replacing fat and salt with edible mushroom flour (*Agaricus bisporus* and *Pleurotus ostreatus*) in frankfurter-type sausages had on physicochemical, microbiological and sensory parameters during cold storage. This reformulation strategy using edible mushroom flour is presented as an interesting substitute for fat and salt and even for meat. It improves the nutritional profile of the sausage, and the physicochemical and sensory properties were affected by the fungus, which gave it a strong umami flavour. Banerjee et al., (2020) [6] evaluated the impact of different amounts (2%, 4% and 6%) of enoki (*Flammulina velutipes*) mushroom stem waste powder on colour, texture, oxidative stability, sensory attributes and the shelf-life of goat meat nuggets. Based on the results of this study, Enoki stem waste extract can be used as a functional ingredient to improve the nutritional profile, physicochemical properties and shelf-life of meat products.

Following this same approach, Madane et al. [7] studied the efficacy of Moringa flower plant extract (MF) to develop a functional chicken product. The incorporation of this extract into chicken meat nuggets improved cooking performance and dietary fibre content without affecting the acceptability of the meat product and increased the lipid stability, odour score and shelf-life of chicken nuggets during refrigerated storage. The authors thus conclude that the MF extract could be used as a safe and natural antioxidant for the meat industry, which also offers functional health-promoting benefits.

Graso et al. [8] tested different concentrations (2 and 4%) of a potential sunflower seed by-product (obtained from sunflower oil extraction) as a substitute for animal fat to develop healthier sausages. The incorporation of this by-product led to a significant increase in protein, minerals (magnesium, potassium, copper, and manganese) and phenolic compounds, and lowered fat content (~37% less than the control made with animal fat). These results show that the use of this ingredient is a viable strategy to enhance the utility of sunflower oil by-products and obtain healthier sausages.

Franco et al. [9] also studied the substitution of pork fat in frankfurters, but in this case used linseed oleogel as the reformulation strategy. This oleogel substantially improved the fatty acid profile, SFA content and the n-6/n-3 ratio, although sensory and lipid oxidation parameters were significantly modified pointing to the need for further study.

Pintado et al. [10] presented a dual study. They first reduced fat by reformulating fresh sausages (longanizas) with chia (*Salvia hispanica* L.) or oat (*Avena sativa* L.) emulsion gel as animal fat replacers. They then studied the effect that cooking procedures (grilling) had on the composition and technological properties of longanizas to determine how nutritional and health claims were affected by this cooking process. After cooking, longanizas prepared with emulsion gels maintained their composition, allowing them to make nutritional and health claims in accordance with European legislation.

Lastly, this SI features two reviews. One [11] presents an interesting bibliographic search on fruit extracts and agro-industrial waste [Jabuticaba (*Myrciaria cauliflora*), Grape (*Vitis* sp.) and Nopal (*Opuntia ficus-indica*)] as sources of compounds potentially acting as antimicrobial agents. These extracts have an important application in meat products as they improve stability and are an interesting alternative to synthetic preservatives. The reduction or replacement of the more traditional preservatives that can be achieved with these extracts also provides products with cleaner labels, a growing trend in the reformulation of healthier compounds where sensory and technological properties remain intact without compromising safety. Use of these by-products also reduces environmental impact. Hence, the search for plant extracts with antimicrobial activity is an area of study on the rise for use in all types of food. The other review by Ruiz-Capillas and Herrero [1] stresses the importance of developing healthier meat products at all stages of the food chain (products, industry, consumers, administration, health organizations, etc.). It focuses mainly on reformulation in relation to healthier lipid content and lipid profile as this is one of the food components that has received the most attention. It also studies the different strategies used to that end such as the novel strategy of forming lipid materials based on structured lipids such emulsion gels (EGs) or oil-bulking agents (OBAs) that offer attractive applications in the reformulation of health-enhanced meat products. The review also conducts a critical analysis of the use of vibrational spectroscopy as a tool to further these developments. This interesting tool helps us gain a better understanding of the structural characteristics of lipid materials (EGs or OBAs) and of the corresponding reformulated health-enhanced meat products into which these fat replacers have been incorporated. These insights help in selecting the best lipid material to achieve specific technological properties in healthier meat products with improved lipid content and acceptable characteristics.

Meat and meat products are extensively consumed throughout the world and make an important nutritional contribution to our diet. However, their consumption has also been associated with some negative health consequences due to some of their components. This has driven significant growth in the field of design and development of healthy meat products focusing mainly on improving their composition, which in turn improves the diet-health relationship of these products. Different strategies have been used at different stages of the farm to fork journey to produce healthier products based on various compounds offering health benefits and featuring appropriate and safe technological and organoleptic properties, which are as similar to those of the original meat product as possible. It should also be noted that any strategy used to produce new healthy meat products will have an effect on the final product, which must be determined and controlled in different ways.

Today there is a great need to swiftly develop heathier meat and meat products to satisfy the demands of consumers who are increasingly aware of the link between diet and health and to comply with health recommendations made by different agencies. Science also needs to be a part of industrial processing so that scientific research can impact consumers in the form of optimal quality products.

The different strategies included in this SI are of interest to all those involved in meat and meat product industry from farm to fork. Lastly, many of the studies featured in this SI leave the door open to future research in this area of growing interest, not only for the meat and meat product industry, but for other foods as well.

## References

[1] Ruiz-Capillas C., Herrero A.M. (2021). Development of Meat Products with Healthier Lipid Content: Vibrational Spectroscopy. Foods.

[2] Núñez-Sánchez N., Ramírez C.A., Blanco F.P., Gómez-Cortés P., De La Fuente M.Á., Amor M.V., Ibáñez A.H., Marín A.L.M. (2021). Effects of Algae Meal Supplementation in Feedlot Lambs with Competent Reticular Groove Reflex on Growth Performance, Carcass Traits and Meat Characteristics. Foods.

[3] Feng Y.H., Zhang S.S., Sun B.Z., Xie P., Wen K.X., Xu C.C. (2020). Changes in Physical Meat Traits, Protein Solubility, and the Microstructure of Different Beef Muscles during Post-Mortem Aging. Foods.

[4] Mahachi L.N., Rudman M., Arnaud E., Muchenje V., Hoffman L.C. (2020). Application of Fat-Tailed Sheep Tail and Backfat to Develop Novel Warthog Cabanossi with Distinct Sensory Attributes. Foods.

[5] Cerón-Guevara M.I., Rangel-Vargas E., Lorenzo J.M., Bermúdez R., Pateiro M., Rodríguez J.A., Sánchez-Ortega I., Santos E.M. (2020). Reduction of Salt and Fat in Frankfurter Sausages by Addition of *Agaricus bisporus* and *Pleurotus ostreatus* Flour. Foods.

[6] Banerjee D.K., Das A.K., Banerjee R., Pateiro M., Nanda P.K., Gadekar Y.P., Biswas S., McClements D.J., Lorenzo J.M. (2020). Application of Enoki Mushroom (*Flammulina velutipes*) Stem Wastes as Functional Ingredients in Goat Meat Nuggets. Foods.

[7] Madane P., Das A.K., Pateiro M., Nanda P.K., Bandyopadhyay S., Jagtap P., Barba F.J., Shewalkar A., Maity B., Jose M. (2019). Drumstick (*Moringa oleifera*) Flower as an Antioxidant Dietary Fibre in Chicken Meat Nuggets. Foods.

[8] Grasso S., Pintado T., Pérez-Jiménez J., Ruiz-Capillas C., Herrero A.H. (2020). Potential of a Sunflower Seed By-Product as Animal Fat Replacer in Healthier Frankfurters. Foods.

[9] Franco D., Martins A.J., López-Pedrouso M., Purriños L., Cerqueira M.A., Vicente A.A., Pastrana L.M., Zapata C., Lorenzo J.M. (2019). Strategy towards Replacing Pork Backfat with a Linseed Oleogel in Frankfurter Sausages and Its Evaluation on Physicochemical, Nutritional, and Sensory Characteristics. Foods.

[10] Pintado T., Ruiz-Capillas C., Jiménez-Colmenero F., Herrero A.M. (2020). Impact of Culinary Procedures on Nutritional and Technological Properties of Reduced-Fat Longanizas Formulated with Chia (*Salvia hispanica* L.) or Oat (*Avena sativa* L.) Emulsion Gel. Foods.

[11] Gonçalves L.A., Lorenzo J.M., Trindade M.A. (2021). Fruit and Agro-Industrial Waste Extracts as Potential Antimicrobials in Meat Products: A Brief Review. Foods.

